# Genome-wide target analysis of NEUROD2 provides new insights into regulation of cortical projection neuron migration and differentiation

**DOI:** 10.1186/s12864-015-1882-9

**Published:** 2015-09-05

**Authors:** Efil Bayam, Gulcan Semra Sahin, Gizem Guzelsoy, Gokhan Guner, Alkan Kabakcioglu, Gulayse Ince-Dunn

**Affiliations:** Molecular Biology and Genetics Department, Koç University, Rumeli Feneri Yolu, Istanbul, 34450 Turkey; Present Address: Neuroscience Department, Washington State University, Pullman, WA 99164 USA; Physics Department, Koç University, Rumeli Feneri Yolu, Istanbul, 34450 Turkey

**Keywords:** NEUROD2, Cortical projection neuron, Cortical layers, Radial migration, Axon guidance

## Abstract

**Background:**

Cellular differentiation programs are controlled, to a large extent, by the combinatorial functioning of specific transcription factors. Cortical projection neurons constitute the major excitatory neuron population within the cortex and mediate long distance communication between the cortex and other brain regions. Our understanding of effector transcription factors and their downstream transcriptional programs that direct the differentiation process of cortical projection neurons is far from complete.

**Results:**

In this study, we carried out a ChIP-Seq (chromatin-immunoprecipitation and sequencing) analysis of NEUROD2, an effector transcription factor expressed in lineages of cortical projection neurons during the peak of cortical excitatory neurogenesis. Our results suggest that during cortical development NEUROD2 targets key genes that are required for Reelin signaling, a major pathway that regulates the migration of neurons from germinal zones to their final layers of residence within the cortex. We also find that NEUROD2 binds to a large set of genes with functions in layer-specific differentiation and in axonal pathfinding of cortical projection neurons.

**Conclusions:**

Our analysis of *in vivo* NEUROD2 target genes offers mechanistic insight into signaling pathways that regulate neuronal migration and axon guidance and identifies genes that are likely to be required for proper cortical development.

**Electronic supplementary material:**

The online version of this article (doi:10.1186/s12864-015-1882-9) contains supplementary material, which is available to authorized users.

## Background

The neocortex is a six-layered structure composed of myriad excitatory and inhibitory neuron types. Developmental programs, controlled by multiple cell-intrinsic and –extrinsic cues, establish unique patterns of connectivity for the diverse neuronal subtypes located in different layers. While the granular neurons of layer IV form networks of local connections within cerebral hemispheres, projection neurons of layers II/III, V and VI mediate long distance communications both within and outside the cortex.

Developmentally, excitatory neurons are born from dividing progenitors located in distinct germinal domains situated at ventricular and subventricular zones of the dorsal telencephalon. Once born, they migrate to superficial cortical layers, with each wave of migrating neurons surpassing predecessors, resulting in six distinct cortical layers generated in an inside-out manner [1]. Consequently, birthdate of a neuron determines its final layer of residence and confers its layer-specific properties. During and following migration, cortical projection neurons initiate expression of specific sets of axon guidance molecules that allows pathfinding to correct targets [2]. The end product of this highly complex and regulated migration and axon guidance process is a neocortex organized into six layers, with neurons residing in each layer exhibiting unique physiological properties and patterns of connectivity [3].

Almost all aspects of neurogenesis, migration and neuronal differentiation are orchestrated by hierarchical and combinatorial functioning of transcription factor (TF) networks [4]. Within the embryonic germinal zones, expression of *Neurog1* and *Neurog2* proneural basic helix loop helix (bHLH) transcription factors commit neural progenitors to cortical excitatory neuron identity [5, 6]. NEUROG2 is both necessary and sufficient for generation of these neurons [7] and NEUROG2 initiated specification of projection neuron identity involves a complex transcription factor network composed of effector transcription factors, such as TBR1 (T-box brain 1), NEUROD1 (Neuronal differentiation 1) and NEUROD2 (Neuronal differentiation 2) [7–9].

Despite our relatively thorough understanding of the TFs required for specifying a cortical excitatory neuron identity, little is known about how differentiation programs are executed by effector TFs and the nature of the downstream target genes required for corticogenesis. For example, *Neurod2* is expressed within a wide temporal window (from embryonic day 10.5 throughout adulthood) outside of the proliferative zones and constitutes a potential regulator of critical aspects of differentiation and/or maintenance of different types of cortical excitatory neurons [8, 10]. Indeed, several studies investigating the consequences of NEUROD2 loss-of-function in mice have demonstrated that NEUROD2 is required for commissural axon pathfinding of layer II/III callosal projection neurons, formation of cortical somatosensory maps within layer IV granular neurons and maturation of dendrites and synapses in the hippocampus [11–13]. Furthermore, a gain-of-function study has demonstrated that misexpression of *Neurod2* in ventral telencephalon progenitors is sufficient to prevent their normal GABAergic differentiation [14].

Although during development NEUROD2 controls the execution of a wide range of functions, its target genes during cortex development are largely unknown. In this study we identified genome-wide targets of NEUROD2 during mid-embryogenesis. We demonstrate that NEUROD2 binds to a large number of target genes with prominent roles in radial migration, layer-specific differentiation and axon pathfinding of cortical projection neurons. Moreover, we find that NEUROD2 is positioned to control cortical radial migration by regulating members of the Reelin signaling pathway. Importantly, we demonstrate that expression of *Cdk5r1*, the regulatory subunit of cyclin-dependent kinase 5, is critically dependent on NEUROD2. Our identification of NEUROD2 targets offers mechanistic insight into known and potentially novel functions of this effector transcription factor and points to the importance of carrying out ChIP-Seq analysis at developmental timepoints within relevant tissue.

## Results and discussion

### Identification of NEUROD2 binding sites in embryonic cortex

**Fig. 1:**
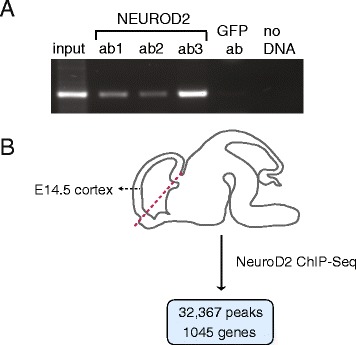
ChIP-Seq reveals NEUROD2 target genes during cortical development. **a** Chromatin DNA was immunoprecipitated with three different antibodies against NEUROD2 (ab1, ab2 or ab3) or with a GFP antibody which was used as a negative control. PCR was performed using immunoprecipitated DNA as template. Specific primers amplified the promoter region of a previously identified NEUROD2 target gene *Nhlh2* [16]. An enrichment for the positive control *Nhlh2* gene was demonstrated in NEUROD2 ChIP-PCR samples as compared to GFP ChIP-PCR. **b** The developing cortex (dorsal telencephalon) was dissected from E14.5 mouse brain and used for NEUROD2 ChIP-Seq. 32,367 peak regions and 1,045 target genes were identified

In mice, the bulk of cortical excitatory neurogenesis takes place during the last ten days of gestation, a period which also coincides with high levels of *Neurod2* expression [10, 15]. Therefore, in order to determine the genome-wide targets of NEUROD2 during cortical development, we carried out chromatin-immunoprecipitation followed by high-throughput sequencing (ChIP-Seq) (Fig. 1b). First, we identified three antibodies that could successfully immunoprecipitate NEUROD2 following overexpression in Neuro2A cell line (Additional file 1). After isolating NEUROD2-associated chromatin from mouse embryonic day 14.5 (E14.5) cortex, we confirmed that the promoter region of the *Nhlh2* gene, a previously identified NEUROD2 target [16], was amplified by PCR using ChIP template DNA precipitated by all three NEUROD2 antibodies, but not from ChIP DNA prepared by an unrelated antibody against GFP (Fig. 1a). After massively parallel sequencing of all three NEUROD2 ChIP DNA and two separate GFP ChIP DNA samples, we mapped reads using Bowtie short read aligner [17, 18]. NEUROD2 peaks were identified by MACS (Model-based analysis of ChIP-Seq) [19] and GFP peak alignments were used as input control dataset for signal normalization (cutoff p-value of < 10^−5^). From the three experimental samples a total of approximately 32,900,000 unique reads were identified, which mapped onto 94,621 peaks (Additional file 2).

The quality of our data was validated by the high degree of correlation among the three NEUROD2 antibodies (Pearson’s correlation coefficient R^2^ values: ab1 and ab2: 0.89; ab1 and ab3: 0.75; ab2 and ab3: 0.84). In contrast, comparison of NEUROD2 peaks generated by the three individual antibodies to those of another transcription factor expressed in the mouse embryonic brain, CTCF, resulted in much lower R^2^ values (ab1 and CTCF ab: −0.16; ab2 and CTCF ab: −0.18; ab3 and CTCF ab: −0.21). In an effort to reduce false-positive binding sites due to non-specific interactions of the particular antibody used, we decided to discard peaks that are identified in only one of the three ChIP-Seq datasets acquired using the three separate antibodies. As a result, out of a total of 94,621 peaks from three separate datasets, 72,871 peaks were found to be located in regions containing overlapping peaks from at least two antibodies. Next, we collapsed all the overlapping peaks to obtain the union of peak coordinates and as a result identified 32,367 NEUROD2 binding locations. Following analyses were carried out using these 32,367 NEUROD2 binding sites (Additional file 3).

**Fig. 2:**
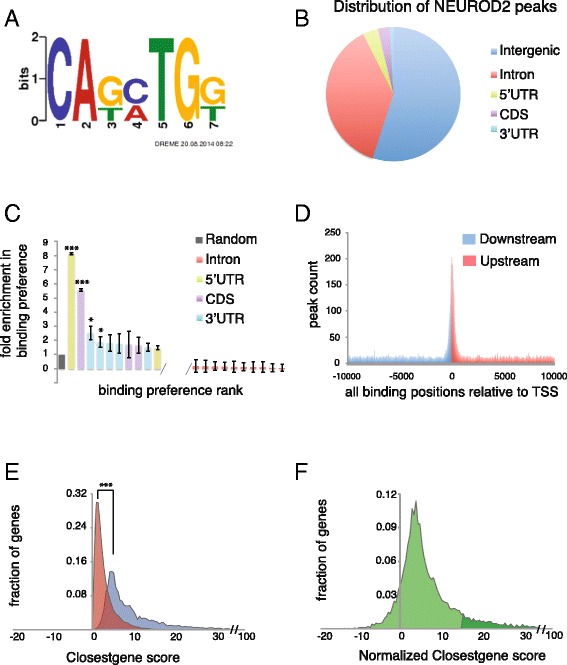
NEUROD2 binds to E-box elements proximal to TSSs *in vivo*. **a** NEUROD2 consensus sequence was predicted using 32,367 peak regions as input for the MEME-ChIP suite [23]. **b** Among the 32,367 NEUROD2 peaks, approximately 45 % mapped onto Ensembl annotated genes (mm10 reference genome) [24, 55]. Among them approximately 83 % mapped onto introns, and 17 % to either 5′UTR, CDS or 3′UTR exons. **c** The binding preference of NEUROD2 to introns, 5′UTR exons, 3′UTR exons and CDS exons was calculated. The number of NEUROD2 binding sites observed on introns (*red*), 5′UTR (*green*), 3′UTR (*blue*) and CDS (*purple*) exons were normalized to the length of each of these gene segments and compared to a random distribution profile (*grey bar*). The data suggests approximately an 8-fold enrichment in NEUROD2 binding to the first 5′UTR exon, a 5-fold enrichment in binding to the first CDS exon and approximately 2-fold enrichment in binding to the first and second 3′UTR exons. ***p-value ~ 0, *p-value < 0.001. **d** A binding map of NEUROD2 relative to closest TSSs, revealed that NEUROD2 has a binding preference within +/− 1000 bps of TSSs. Peaks that mapped upstream (*blue*) and downstream (*red*) of TSSs are color-coded. **e** The distribution profiles of ClosestGene scores [29] calculated by using NEUROD2 peaks (*blue*) were very significantly different from those calculated using randomly distributed peaks (*red*) on the genome (***p-value ~ 0). **f** Distribution of normalized scores for individual genes were generated by subtracting the ClosestGene score calculated using NEUROD2 peaks from that of random peaks. Dark green represents genes whose scores were greater than one standard deviation (*σ*) above the mean and were used for further analyses

Neuronal bHLH transcription factors like NEUROD2 bind to an E-box motif, which is loosely defined as CANNTG [20–22]. Therefore, we asked whether or not the NEUROD2 peak sequences were enriched in E-box elements. By using the MEME-CHIP Suite [23], a tool designed to identify enriched sequence elements in large datasets, we discovered that an extended E-box motif, CA(G/T)(C/A)TG(G/T), was significantly associated with NEUROD2 peak sequences (Fig. 2a).

In order to determine NEUROD2 binding preference along the genome and therefore gain insight into its mechanism of action, we examined peak positions relative to annotated transcriptional units. Analysis of all 32,367 sites revealed that approximately half of NEUROD2 binding locations (45 %) mapped onto annotated Ensembl transcripts (mm10 build) [24] and the remainder mapped onto intergenic regions. Within a gene, 83 % of intragenic sites were associated with intronic regions and 17 % were associated with 5′UTR, coding (CDS), or 3′UTR exons (Fig. 2b). However, on average introns are ~25–30 times longer than exons [25], therefore in order to normalize for this length bias we calculated the probability of binding along a gene compared to a random distribution profile. Our calculations revealed that NEUROD2 binding was most likely to be embedded in the first 5′UTR exon with an approximately 8-fold enrichment in probability compared to random distribution (p-value = 1 × 10^−2132^) (Fig. 2c). Next, we generated a NEUROD2 binding map relative to annotated transcription start sites (TSSs) and observed a major accumulation of NEUROD2 peaks within 1000 bps from a TSS (Fig. 2d). Therefore, *in vivo* NEUROD2 preferentially binds to TSS proximal sequences located within or upstream of 5′UTR exons.

A previous study used a ChIP-Seq approach to investigate genome-wide targets of NEUROD2 after its viral transduction into the P19 cell line [16]. However, since this study was conducted in cell culture, it may not provide an accurate reflection of the *in vivo* targets of NEUROD2 during cortex development. In fact, recent studies propose that transcription factor-chromatin associations are context dependent and can vary between developmental stages and tissue types [26–28]. Therefore, within the context of cortical development, with the exception of a small number of candidate genes studied so far, NEUROD2’s genome-wide targets and the kinds of biological processes regulated are largely unknown [11–13]. Since our data demonstrate that NEUROD2 binding was not exclusive to promoter regions or TSS-proximal sequences, we decided to employ a method for assigning individual peaks to genes which would also account for TSS-distant peaks, as opposed to methods which set fixed windows around TSSs for peak capturing. For this reason, we decided to use the *ClosestGene* method which assigns scores to individual genes based on the number of associated peaks and their proximity to respective transcription start sites [29]. We calculated two scores for each gene in the mouse genome by using either the 32,367 NEUROD2 peaks or the same number of randomly distributed peaks as the input dataset. Plotting the distribution profiles of the scores calculated by NEUROD2 peaks or random peaks revealed a very significant difference between these two datasets (p-value ~ 0) (Fig. 2e). Next, we calculated a normalized ClosestGene score for each gene by simply subtracting the random score from the NEUROD2 score (Additional file 4). We then selected putative target genes, with varying scores, for confirmation by NEUROD2 ChIP followed by quantitative PCR (qPCR) using the immunoprecipitated DNA as template. We selected genes from the top 1,045, representing genes whose scores were above one standard deviation (σ) of the mean and were able to confirm 10 out of 11 (Fig. 2f and Additional file 5). In addition, as negative controls we tested 5 genes (*Gsx2, Dlx2, Npy, Gad1* and *Calb2*) which were not identified as NEUROD2 targets (i.e., non-targets). These five genes are expressed in interneurons and not in *Neurod2*-expressing cortical projection neurons. We did not observe any significant enrichment in NEUROD2 binding to 4/5 of the non-target genes and only a slight enrichment in *Gsx2,* a finding worthy of future investigation (Additional file 5). In sum, in light of our high confirmation rate of NEUROD2 binding to putative target genes we decided to use the top 1,045 in all further analyses.

### NEUROD2 target genes are associated with radial migration of cortical neurons

To further gain broad understanding into the biological processes regulated by NEUROD2 we carried out gene ontology analysis. We identified significantly over-represented biological processes in the top 1,045 targets against all of the genes in the mouse genome. Ranking based on fold enrichment of biological processes above a significance threshold (p-value < 10^−3^), revealed that the highly ranked NEUROD2 controlled activities broadly converged onto two major developmental processes; cortical radial migration and axon guidance/fasciculation (Table 1).

**Table 1:** Gene Ontology Analysis of NEUROD2 targets

Biological process	Fold enrichment	p-value
Neuron projection guidance	3.61	3.36 × 10^−08^
Axon guidance	3.56	8.24 × 10^−04^
Forebrain neuron differentiation	3.15	1.53 × 10^−04^
Forebrain cell migration	2.93	4.91 × 10^−08^
Central nervous system neuron development	2.91	6.79 × 10^−06^
Cerebral cortex cell migration	2.88	2.81 × 10^−09^
Telencephalon cell migration	2.86	1.12 × 10^−07^
Cerebral cortex radially oriented cell migration	2.75	3.3 × 10^−05^
Axon fasciculation	2.69	7.55 × 10^−18^
Neuron recognition	2.69	7.55 × 10^−18^

Biological process categories above a cutoff p-value (<1 × 10^−3^) are ranked by fold enrichment. Fold enrichment is calculated as log_2_(observed/expected)

**Fig. 3:**
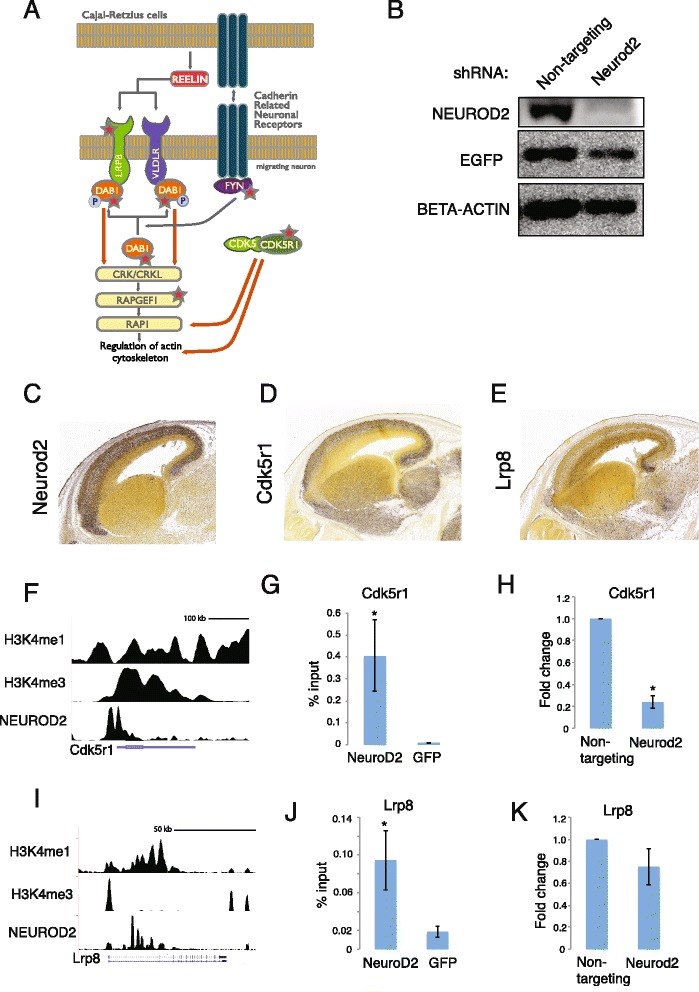
NEUROD2 targets *Cdk5r1* and *Lrp8* genes during corticogenesis. **a** Diagrammatic representation of the Reelin signaling pathway that is required for cortical radial migration. Genes that are targets of NEUROD2 are marked with a red star. **b** NEUROD2 western blotting confirms knockdown in primary cortical neuronal culture transfected with Neurod2 shRNA as compared to a non-targeting shRNA. EGFP is used to verify transfection efficiency and BETA-ACTIN is used as a loading control. **c**, **d** and **e** mRNA expression data is obtained from Allen Developing Mouse Brain Atlas (http://developingmouse.brain-map.org) for *Neurod2*, *Cdk5r1* and *Lrp8* genes in E15.5 mouse brain. **f** and **i** NEUROD2 peaks associated with *Cdk5r1* and *Lrp8* are plotted relative to enhancer (H3K4me1) and promoter (H3K4me3) marks. H3K4me1 and H3K4me3 ChIP-Seq data is from (www.encodeproject.org) [31]. Peaks are plotted using the genome browser at genome.ucsc.edu. **g** and **j** NEUROD2 ChIP followed by qPCR confirms NEUROD2 binding to the promoter regions of *Cdk5r1* and *Lrp8* relative to a negative control (GFP ChIP). Data is normalized to the amount of input DNA as described in the *Methods* section (p-value < 0.05). Bars represent standard error of mean. Data represents two biological and five technical replicates. **h** and **k** Reverse transcription and qPCR analysis of neurons transfected with Neurod2 shRNA or non-targeting shRNA reveals a significant reduction in *Cdk5r1* mRNA levels (p-value = 9.6x10^−8^) and a trend of reduction in *Lrp8* levels (p-value = 0.18) after *Neurod2* knockdown. All RT-qPCR results represented data from 3 biological samples each analyzed in technical triplicates. Bars represent standard error of mean

To gain further understanding of the signaling pathways that were regulated by NEUROD2, we carried out pathway analysis with the NEUROD2 target genes against all pathways present in the Biocarta database (http://www.biocarta.com/genes/index.asp). Interestingly, we identified *Reelin Signaling* as the most significantly targeted pathway (p-value: 4.7 × 10^−3^) (Additional file 6). The REELIN protein is secreted by the Cajal-Retzius cells, located on the surface of the developing cortex. By binding to receptors on migrating cortical neurons, REELIN promotes reorganization of the cytoskeleton and regulates neuronal migration (Fig. 3a) [1, 30]. Our analysis revealed that NEUROD2 targets included several key members of this pathway, such as *Fyn*, *Cdk5r1*, *Dab1* and *Lrp8 (*ApoE Receptor 2) genes (Fig. 3a).

We next focused our analysis on two important regulators of cortical radial migration, *Cdk5r1* and *Lrp8* genes. Initially, examination of expression patterns at mid-embryogenesis (E15.5) revealed that *Neurod2* expression largely overlapped with zones of *Lrp8* and *Cdk5r1* expression (Allen Developing Mouse Brain Atlas, Fig. 3c, d and e). Next, we examined whether NEUROD2 binding along *Cdk5r1* and *Lrp8* genes were associated with enhancer or promoter regions. We acquired ChIP-Seq data from E14.5 whole brain for H3K4me1 histone modification as a marker for enhancer regions and H3K4me3 modification as a marker for promoter regions [31] (www.encodeproject.org). Overlaying of these datasets with NEUROD2 peaks revealed that NEUROD2 binding was associated with both promoter and enhancer specific histone marks on these two genes (Fig. 3f and i). We further confirmed NEUROD2 association with the promoter regions of *Lrp8* and *Cdk5r1* genes by NEUROD2 ChIP followed by qPCR (Fig. 3g and j). Finally, we asked whether NEUROD2 controls *Cdk5r1* and *Lrp8* transcription. We knocked down *Neurod2* expression almost completely in primary cortical neurons by high-efficiency transfection of small hairpin RNA (Fig. 3b). Remarkably, our RT-qPCR results demonstrated a significant, five-fold reduction in *Cdk5r1* mRNA levels after *Neurod2* knockdown, as compared to a control sample transfected with a non-targeting shRNA (Fig. 3h). *Lrp8* mRNA also exhibited a trend in reduction in NEUROD2 lacking neurons, however with lower significance (Fig. 3k). In sum, our results strongly support NEUROD2 control of radial migration and the Reelin signaling pathway through direct regulation of key genes such as *Cdk5r1* and *Lrp8*.

**Fig. 4:**
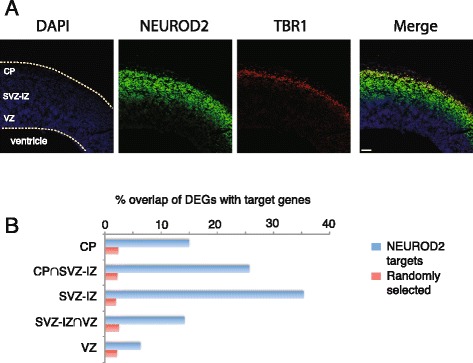
NEUROD2 target genes are enriched in the subventricular-intermediate zone of the developing cortex. **a** Immunofluorescent staining against NEUROD2 and TBR1, and DAPI staining on coronal sections prepared from E14.5 mouse brain. NEUROD2 positive neurons are observed in the SVZ-IZ region of the developing cortex. Scale bar, 62 μm. **b** The percentage of overlap between NEUROD2 target genes and differentially expressed genes (DEGs) in the cortical plate (CP), subventricular zone/intermediate zone (SVZ-IZ), ventricular zone (VZ), and those that are preferentially expressed in two regions (CP∩SVZ-IZ and SVZ-IZ∩VZ) are plotted. NEUROD2 target genes (*in blue*) display an enrichment in DEGs of the different cortical regions (in particular the SVZ-IZ region) as compared to randomly selected genes (*in red*) from the mouse genome

Next, we asked whether NEUROD2 was expressed in neurons of the intermediate zone (IZ), which is a developmentally transient cortical region through which post-mitotic neurons born in subventricular (SVZ) and ventricular (VZ) zones migrate in order to reach their final destination within the cortical plate (CP) [32]. To mark neurons that have completed their migration and are localized to the cortical plate, we performed co-staining with antibodies against the TBR1 transcription factor and NEUROD2. As expected, we identified NEUROD2+/TBR1+ neurons localized to the CP (Fig. 4a). In addition, a large fraction of NEUROD2+ neurons were localized within the IZ, below the layer of TBR1+ CP, most likely constituting the migratory population. Taken together, our results are consistent with a model in which NEUROD2 expression is initiated in post-mitotic neurons right before the onset of migration and regulates a set of genes, that includes critical components of the Reelin pathway.

Next, in an unbiased approach to identify probable gene candidates regulating migration through the IZ, we asked whether the expression of a particular set of NEUROD2 targets were enriched in the neurons of this zone. Towards this goal, we took advantage of a study describing differentially expressed transcripts by laser capture microscopy followed by mRNA-SEQ within three major regions of the E14 cortex; cortical plate (CP), subventricular-intermediate zones (SVZ-IZ) and ventricular zone (VZ) [33]. In this study, each differentially expressed gene (DEG) was categorized as being strictly specific to one zone (CP, SVZ-IZ and VZ), or as being expressed in two of the three zones (CP∩SVZ-IZ and SVZ-IZ∩VZ). A comparison of NEUROD2 targets with the expression profiles of DEGs revealed that approximately 35 % of all SVZ-IZ specific genes (representing 18 genes in total) were also targets of NEUROD2 (p-value < 0.05) (Fig. 4b and Additional file 7). This comparison revealed lower percentages for the other four categories. For example, in the VZ where post-mitotic neurons are excluded and cell division of progenitor neurons takes place, only 6 % of DEGs were also targets of NEUROD2. The overlap between DEGs in any category and equivalent numbers of randomly selected genes from the mouse genome was approximately 3 % (Fig. 4b). Consistent with known functions of previously identified genes that are required for radial migration, the functions of the 18 SVZ-IZ specific NEUROD2 targets converged on axon guidance, regulation of neuronal polarity and actin cytoskeleton (Additional file 7) [1]. Taken together, our combined data strongly support a role for NEUROD2 in radial migration of cortical neurons.

### NEUROD2 is positioned to control differentiation and axonal guidance of distinct cortical projection neuron lineages

**Fig. 5:**
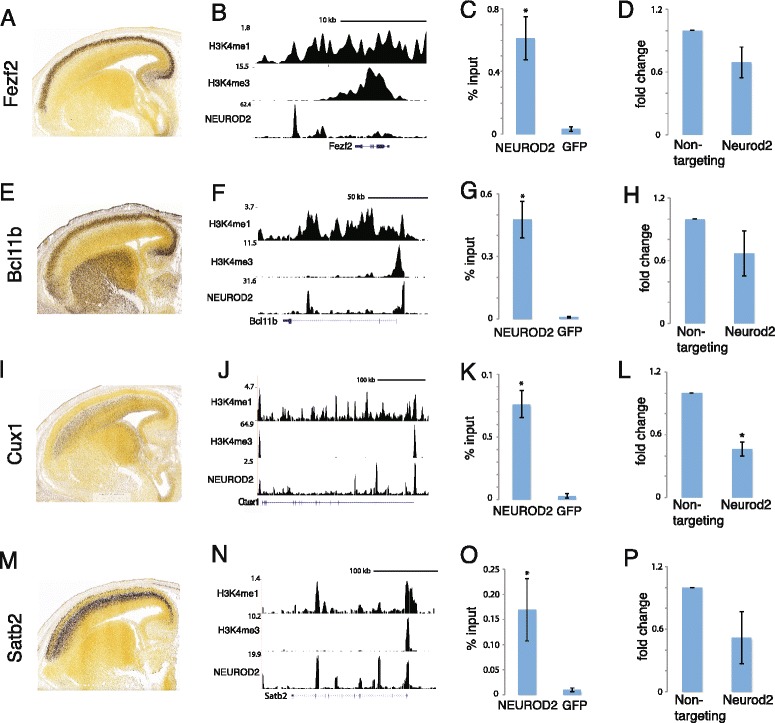
NEUROD2 target genes encode transcription factors that control the differentiation of distinct populations of cortical layer specific projection neurons. mRNA expression data is obtained from Allen Developing Mouse Brain Atlas (http://developingmouse.brain-map.org) for *Fezf2* (**a**), *Bcl11b* (**e**), *Cux1* (**i**) and *Satb2* (**m**) for E15.5 mouse brain. *Neurod2* expression overlaps with the domains expressing each of the cortical layer specific transcription factors. (**b**, **f**, **j** and **n**) NEUROD2 peaks are plotted relative to enhancer (H3K4me1) and promoter (H3K4me3) marks. H3K4me1 and H3K4me3 ChIP-Seq data is from (www.encodeproject.org) [31]. Peaks are plotted using the genome browser at genome.ucsc.edu. (**c**, **g**, **k** and **o**) NEUROD2 ChIP followed by qPCR confirms NEUROD2 binding to the promoter regions of all four layer markers relative to a negative control (GFP ChIP) (p-value < 0.05). Data is normalized to the amount of input DNA as described in the *Methods* section. Bars represent standard error of mean. Data represents two biological and five technical replicates. (**d**, **h**, **l** and **p**) mRNA levels of *Fezf2, Bcl11b, Cux1* and *Satb2* were quantified by RT-qPCR in neurons transfected with either a non-targeting or a Neurod2 shRNA. A significant reduction in *Cux1* mRNA levels was observed in the absence of NEUROD2. Each experiment represents 3 biological samples each analyzed in technical triplicates. Data is presented as fold change of control sample (* p-value = 0.0014). p-values for changes in expression of *Fezf2*, *Bcl11b* and *Satb2* were between 0.1 and 0.2. A non-targeting shRNA was used as control. Bars are standard error of the mean

Neurons that are migrating through the IZ during mid-embryogenesis differentiate into different types of cortical projection neurons [15]. The genetic programs that specify the identities of distinct cortical projection neuron lineages are only partially understood. Loss-of-function studies of a set of layer-specific transcription factors have unveiled their requirement for distinct axon guidance programs and establishment of lamina-specific connectivity [2, 34–38]. Interestingly, we noticed the presence of such a group of transcription factors that control the differentiation of layer-specific properties of cortical projection neurons in our NEUROD2 target gene dataset. We identified that both deep layer markers, such as *Fezf2* and *Bcl11b (Ctip2)* and upper layer markers such as *Cux1* and *Satb2* scored highly in our NEUROD2 target gene list (Additional file 4). Analysis of NEUROD2 peaks that mapped onto the genetic loci of *Fezf2*, *Bcl11b*, *Cux1* and *Satb2* uncovered association at several distinct points which overlapped with enhancer (H3K4me1) and promoter (H3K4me3) specific histone marks (Fig. 5b, f, j and n). Examination of expression patterns at mid-embryogenesis revealed that *Neurod2* expression overlapped with the regions expressing *Fezf2*, *Bcl11b*, *Cux1* and *Satb2* (Allen Developing Mouse Brain Atlas and Fig. 5a, e, i, and m).

Next, we confirmed NEUROD2 association with the promoter regions of these genes, by NEUROD2 ChIP coupled with quantitative PCR (Fig. 5c, g, k and o). Our analysis revealed that NEUROD2 associated strongly with the promoter regions of *Fezf2*, *Bcl11b*, *Cux1* and* Satb2 *genes. In order to determine whether NEUROD2 is required for the expression of cortical layer-specific transcription factors, we quantified their mRNA levels by RT-qPCR after Neurod2 knockdown in primary cortical neurons (Fig. 5d, h, l, and p). Interestingly, we observed a general trend in which all four transcription factor levels were reduced, albeit at varying levels of significance. In particular, the expression of *Cux1* mRNA was very significantly decreased by approximately 60 %. Together, our results argue that NEUROD2 is a principal regulator of a group of transcription factors that confer layer-specific identities to cortical projection neurons.

Given that a major function of layer-specific transcription factors during cortical development is to express the correct set of axon guidance receptors and establish proper connectivity, next we focused on how NEUROD2 might be involved in this process. Evidence supporting this possibility comes from a study demonstrating that *Neurod2* genetically interacts with another bHLH neuronal differentiation factor, *Neurod6*, to establish the corpus callosum and the anterior commissure fiber tract [11]. These axon guidance defects have been correlated with dysregulation of the cell adhesion protein *Cntn2* and the axonal receptor *Robo1* in *Neurod2/6* double knockout mice [11]. To our knowledge no other study has investigated a role for NEUROD2 in fasciculation and guidance of cortical projection neuron axons. Consequently, the full extent of the NEUROD2 regulated axon guidance pathways is unknown.

Our previous pathway analysis using the Biocarta database identified the Reelin signaling pathway as a primary target of NEUROD2, however did not reveal any suggestions of NEUROD2 regulation of cortical neuronal connectivity. Since different pathway databases have the potential to illuminate different aspects of a given gene set, to further explore NEUROD2 controlled pathways we compared our set of target genes against the KEGG (Kyoto Encyclopedia of Genes and Genomes) pathway database using David Bioinformatics Resources [39, 40]. Interestingly, our pathway analysis revealed *axon guidance* as the most significantly targeted pathway (p-value: 4.2 × 10^−13^) (Additional file 6). Among the 132 genes which construct different axon guidance pathways in the KEGG database, 30 were targets of NEUROD2 (Table 2 and Additional file 8). In particular, we observed an enrichment for genes that constitute the Ephrin/Eph Receptor, Semaphorin/Plexin Receptor and Slit/Robo Receptor signaling pathways. Our results are consistent with a role for NEUROD2 in callosal axon guidance and in formation of commissural fiber tracts via direct expression of receptors or ligands mediating axon repulsion and attraction during the mid-embryogenic period.

**Table 2:** Top ten axon guidance genes targeted by NEUROD2

Gene symbol	Gene name	Ensembl ID	Normalized ClosestGene score
Epha3	Eph receptor A3	ENSMUST00000064405	52.6
Plxna4	Plexin A4	ENSMUST00000115096	51.8
Slit1	Slit homolog 1	ENSMUST00000025993	48.8
Plxna2	Plexin A2	ENSMUST00000027952	46.9
Sema5b	Semaphorin 5B	ENSMUST00000120756	42.3
Epha4	Eph receptor A4	ENSMUST00000027451	39.9
Nfatc2	Nuclear factor of activated T-cells	ENSMUST00000109184	39.6
Robo2	Roundabout homolog 2	ENSMUST00000023600	36.1
Unc5a	Unc-5 homolog A	ENSMUST00000109994	34.7
Robo1	Roundabout homolog 1	ENSMUST00000023600	33.0

In this study, we have identified the *in vivo* targets of NEUROD2, an effector transcription factor during the peak of cortical excitatory neurogenesis in mice. We have generated our NEUROD2 target gene dataset by carrying out a ChIP-Seq analysis on the cortex region of E14.5 stage mouse embryos. We discovered an enrichment of NEUROD2 binding locations at TSSs. However, we also observed that many binding sites were distant to TSSs and overlapped with enhancer regions. Our observations were in agreement with recent evidence suggesting that TSS-distal transcription factor binding sites are critical regulators of cell-type specific gene expression programs [41–44]. Therefore, to assign binding locations to individual genes, we utilized the ClosestGene method in order to capture the TSS-proximal and –distal peaks. Following our computational analysis we predicted 1,045 NEUROD2 target genes and verified a selected subset at a high rate of confirmation (10/11).

We found that NEUROD2 gene targets include critical regulators of radial migration of cortical neurons from the ventricular and subventricular zones to the cortical plate. A precisely regulated migration process in the developing cortex is essential for the establishment of a six-layered cortex. Molecularly, cortical radial migration requires REELIN, a glycosylated extracellular protein that is secreted by the Cajal-Retzius cells located in the most superficial marginal zone of the developing cortex. Reelin binds to LRP8 (ApoER2) and VLDLR receptors that are expressed by migrating cortical neurons and by doing so activates downstream signaling pathways that eventually reorganize the cytoskeleton and promote migration [1, 45]. Our data revealed that NEUROD2 binds to *Lrp8* gene at regions overlapping with promoter and enhancer specific histone markers and also possibly regulates its expression. Other essential regulators of cortical radial migration are the serine-threonine kinase CDK5 (cyclin dependent kinase 5) and its regulatory subunit CDK5R1 (p35). Loss-of-function mutations of both CDK5 and CDK5R1 result in impaired radial migration and abnormal cortical lamination [1, 46, 47]. Intriguingly, we demonstrated that NEUROD2 binds to and regulates the expression of *Cdk5r1* gene in cortical neurons. To date, a direct functional role for NEUROD2 in cortical migration has not been tested. However, consistent with our findings, a recent study reports abnormal laminar positioning of cortical projection neurons in *Neurod2* knockout mice [11]. Looking forward we are greatly interested in directly testing a role for NEUROD2 in the cortical migration process.

Consistent with our gene ontology results we showed that NEUROD2 was expressed in the neurons of the SVZ and IZ, which are zones of migrating neurons. Further, overlaying our dataset with a study describing differentially expressed genes in functionally distinct zones of the embryonic cortex revealed that NEUROD2 is associated with approximately 35 % of all genes enriched in the SVZ and IZ [33]. Many of these genes have not been investigated with regard to their functions in radial migration, and are highly likely to have essential roles in this process as they encode proteins that organize the neuronal cytoskeleton and control cellular adhesion. We also observed a number of genes that regulate axonal pathfinding within this overlapping dataset, including *Epha3, Plxna2* and *Robo2*, which might indicate additional roles for these receptors in migration, as it has been recently suggested [48–51]. In sum, our study is in agreement with a model in which NEUROD2 expression is initiated in post-mitotic cortical neurons and regulates their migration through the transcription of a set of genes controlling actin and microtubule dynamics and cell surface molecules with roles in cellular adhesion.

Finally, our data suggested that in addition to regulating the migration of cortical projection neurons, NEUROD2 also targets transcription factors that control the layer-specific identities and connectivity patterns of neurons located in different layers. In particular, we provided evidence that NEUROD2 controls both deep-layer markers, such as *Fezf2* and *Bcl11b (Ctip2)*, as well as upper-layer markers such as *Satb2* and *Cux1*. In summary, our dataset offers an exciting list of genes and pathways with putative roles in migration, layer-specific differentiation and axonal migration.

## Conclusions

Significant progress in recent years has identified critical transcription factors which specify progenitors to the cortical projection neuron fate. However, little is known about the effector programs that further control the differentiation of this important neuronal subtype [3, 15]. In this study, we report genome-wide targets of an effector transcription factor, NEUROD2, using a ChIP-Seq approach. Our results demonstrate that in mice at mid-embryogenesis (E14.5), a time point which overlaps with the peak of cortical excitatory neurogenesis, NEUROD2 binds to a set of genes whose functions coalesce into two essential processes; radial migration and axon guidance. Prominently, we verify that expression of *Cdk5r1*, a critical regulator of radial migration, and *Cux1*, a transcription factor which controls the differentiation of upper layer (II/III and IV) projection neurons [52–54] is dependent on NEUROD2. Our study highlights the value of ChIP-Seq experiments conducted within relevant tissue and during a developmental period [16] and offers the potential of unveiling novel genes and pathways regulating cortical projection neuron differentiation. NEUROD2 controls the differentiation and physiology of a variety of different types of neurons such as those located in the hippocampus, amygdala and cerebellum. Looking forward, it is of interest to identify NEUROD2 targets in the context of different spatiotemporal settings within the developing nervous system.

## Methods

### Chromatin immunoprecipitation (ChIP) and sequencing

Mouse embryonic day 14.5 cortices were dissected in ice-cold 1x HBSS containing 100 mM HEPES. For each ChIP-Seq experiment 30 embryos derived from 5 pregnant mothers were used. After dissection, tissue was triturated once in 1x HBSS and cross-linked for 10 min. in 1 % formaldehyde. Cross-linking was quenched by addition of glycine to 125 mM final concentration for 10 min. Tissue was lysed in cold RIPA buffer (0.05 M Tris–HCl pH 7.5, 0.15 M NaCl, 1 % Triton-X 100, 1 % Na-DOC, 0.1 % SDS) containing freshly added protease inhibitors and sonicated using a Bandelin Sonoplus HD2070 sonicator for 30 times at 70 % output for 20 s in the cold-room. Pellets was discarded after a 20 min. 4 °C centrifugation step at 14,000 rpm. Lysates was pre-cleared in Protein A/G magnetic beads (Pierce, cat. 88803). 10 % of the pre-cleared lysate was set aside as input control. Pre-cleared lysates were transferred to fresh eppendorfs and incubated with Protein A/G beads and ChIP antibodies (NEUROD2 antibodies are from Abcam, cat. ab104430, ab109406, ab168932; GFP antibodies are from Santa Cruz, cat. sc-8334, and Pierce, cat. 4B10B2). All antibodies were used at a 10 μg/ml final concentration. After antibody incubations beads were washed with seven different wash buffers twice for 10 minutes. (Buffer 1: 1X PBS, 0.1 % SDS, 0.5 % Na-DOC, 0.5 % NP-40; Buffer 2: 5X PBS, 0.1 % SDS, 0.5 % Na-DOC, 0.5 % NP-40; Buffer 3: 15 mM Tris–HCl, pH 7.5, 5 mM EDTA, 2.5 mM EGTA, 1 % Triton X-100, 1 % Na-DOC, 0.1 % SDS, 120 mM NaCl, 25 mM KCl; Buffer 4: 15 mM Tris–HCl, pH 7.5, 5 mM EDTA, 2.5 mM EGTA, 1 % Triton X-100, 1 % Na-DOC, 0.1 % SDS, 1 M NaCl; Buffer 5: 15 mM Tris–HCl, pH 7.5, 5 mM EDTA; Buffer 6: 50 mM Tris–HCl, pH 7.5, 150 mM NaCl, 1 mM MgCl_2_, 0.05 % NP-40; Buffer 7: 50 mM Tris–HCl, pH 7.5, 10 mM MgCl_2_, 0.5 % NP-40). ChIP DNA was eluted in 300 μl elution buffer 1 (1 % SDS, 0.1 M NaHCO_3_) at 65 °C for an hour. Beads were discarded and the eluate was incubated for an additional 12–15 h at 65 °C. Equal volume of elution buffer 2 (100 mM Tris–HCl, 20 mM EDTA pH 8.0) and 1 μl of RNAse A (Thermo Scientific, cat. R1253) was added and incubated at 37 °C for 1 h. Then, 4 μl of Proteinase K (Thermo Scientific, cat. EO0491) was added and incubated at 50 °C for 2 h. ChIP DNA was purified by standard phenol-chloroform extraction followed by ethanol precipitation. Finally, library preparation and 50 bp single end sequencing (Solexa HiSeq 2500 platform) services were performed at Genewiz, Inc., USA.

### Data access

The data from this study were submitted to Gene Expression Omnibus (GEO) under the accession number [GEO:GSE67539].

### ChIP and qPCR of NEUROD2 binding sites

Ten percent of the lysate used for a single ChIP experiment was set aside as input. Using this input, chromatin DNA was prepared by standard phenol-chloroform extraction and ethanol precipitation. Input (input DNA) and chromatin immunoprecipitated DNA (ChIP DNA) were each dissolved in 300 μl water and 1 μl of each was used in a single qPCR reaction. The numerical value 3.32 (log_2_10, representing 10 % of input chromatin) was subtracted from the Ct value of the input sample to generate the adjusted input Ct. Following formula was used to calculate the input normalized chromatin immunoprecipitated DNA amount: 100 × 2^(Adjusted input Ct – ChIP Ct)^. ChIP DNA was prepared using NEUROD2 antibody 2 and negative control ChIP DNA by using GFP antibody 2 for all qPCR experiments (see Additional file 2). Primer sequences are provided in Additional file 9.

### Neurod2 knockdown in primary cortical neurons

An shRNA targeting Neurod2 was cloned into pSUPER-neo-GFP backbone vector as described in oligoengine.com. Oligos used for cloning are provided in Additional file 9. Primary cortical neuronal cultures were prepared from E14.5 pups. shRNA encoding plasmids were transfected into primary cortical neurons immediately before plating by nucleofection (P3 primary cell 4-D nucleofector X kit, Lonza, program no: CU-133). After plating, transfected neurons were cultured for 4 days *in vitro* before harvesting. For western blotting cells were lysed in ice-cold RIPA buffer. For total RNA isolation cells were lysed in Trizol (Thermo Scientific, Inc.).

### Reverse transcription and qPCR of NEUROD2 target genes

Ten nanogram total RNA was used to prepare cDNA from Neurod2 knockdown and non-targeting samples (Transcriptor high-fidelity cDNA synthesis kit, Roche). mRNA levels were quantified by qPCR analysis (Luminaris Color Higreen qPCR Master Mix, Thermo Scientific) with primers listed in Additional file 9 (CFX Connect Real-Time PCR Detection System, BioRad).

### Identification of NEUROD2 peaks

Peak calling was performed using tools available on Galaxy Project (usegalaxy.org). Tool names and versions are indicated below. Single end 50 bp raw sequences in fastq format were mapped onto the mouse genome (mm10 build) with *Bowtie for Illumina* (version 1.1.2) [17, 18]. Two mismatches were allowed within a 28 nt seed region. PCR duplicates with identical start and end points were removed and collapsed onto a single read with *rmdup* (version 1.0.0). Reads that mapped onto multiple locations on the genome were removed with *Filter SAM* (version 1.0.0). Peaks locations were identified with *MACS* (Model-based analysis of ChIP-Seq version 1.0.1) [19]. 50 nt tag size, 200 nt band width, 1 × 10^−5^ p-value cutoff and an MFOLD high confidence enrichment ratio of 20 were used as input parameters. Results from ChIP performed using GFP antibodies were used as ChIP-Seq control files for MACS analysis. GFP ChIP reads generated with a rabbit antibody (Santa Cruz cat. sc-8334) were used as input controls for NEUROD2 ChIP reads generated with rabbit antibodies (Abcam cat. ab104430 and ab109406). GFP ChIP reads generated with a mouse antibody (Pierce, cat. 4B10B2) were used as input controls for NEUROD2 ChIP reads generated with a mouse antibody (Abcam cat. ab168932). The antibody information is presented in Additional file 2.

### Bioinformatics analysis of peak sequences

MACS outputs from ChIP-Seq data using different NEUROD2 antibodies were combined to yield a single set of high-confidence peak locations. To this end, we first eliminated isolated instances where a MACS peak from an antibody has no overlap with those derived from the other two antibodies. Remaining overlapping peaks were next merged to yield a single set of 32,367 peak regions which, by construction, are confirmed by at least two experiments. The midpoint of each peak was used as the peak location.

The statistical analysis of peak locations was done on the genome assembly GRCm38/mm10 with Ensembl annotations for coding and noncoding regions CDS, 5′UTRs, 3′UTRs, and introns [24, 55]. In order to prevent double counting due to multiple transcripts per gene in the Ensembl database, the longest transcript was selected as representative of each gene. All numerical analyses were performed by means of custom Perl scripts using the Bioperl module for efficiency.

Binding preference of NEUROD2 to different types of intragenic regions was investigated by comparing the observed peak count *n* for a given region type with the corresponding mean $\overline{n}$ and standard deviation *σ*, both of which were obtained from a uniformly random reassignment of the observed intragenic peaks to transcript regions genome-wide. We measured the binding preference in terms of the deviation of the observed peak count from the random expectation, where the deviation was expressed in multiples of the standard deviation, i.e., $bp=\left(n-\overline{n}\right)/\sigma .$

Affinity of NEUROD2 for binding specifically in the proximity of transcription start sites (TSSs) was investigated by considering all peak/TSS pairs located up to 10^6^ bps apart and constructing the corresponding peak-TSS distance histogram.

NEUROD2 consensus sequence was determined by using MEME ChIP Suite using 32,367 NEUROD2 peak sequences as input. Accession numbers for data downloaded from Encode Project (encodeproject.org) were: For E14.5 whole brain ChIP using antibodies against, H3K4me1 (ENCSR000CCZ), H3K4me3 (ENCSR000CDA) and CTCF (ENCSR000CEH) [31]. All data downloaded from Encode Project were generated by the Bing Ren Laboratory, UCSD. Gene ontology analysis was performed as described in [56].

### Target gene identification

Individual peaks were assigned to genes using the *ClosestGene* method as described in [29]. Briefly, each of the 32,367 NEUROD2 peaks was assigned to the closest transcription start site belonging to a specific gene and received a positive score that decreased with the distance from the assigned TSS. Each gene received a cumulative *ClosestGene* target score which was the sum of the scores of all associated peaks. As a background control 32,367 randomly distributed peaks along the mouse genome were assigned to specific genes using the same method.

### Use of animals

All mice were bred and housed at Koç University Animal Research Laboratory. Timed pregnant BALB/c strain mice at E14.5 stage were sacrificed by cervical dislocation, and embryos were retrieved into ice-cold 1x HBSS for cortical dissection. All animal experiments were done in accordance with guidelines provided by the Koç University, The Turkish Ministries of Food, Agriculture and Live Stock, Forestry and Water Management and the European Union. Ethics approval was obtained from The Koç University Institutional Animal Care and Use Committee (no. 2013–1).

## Additional files

Additional file 1:
**In this file we provide raw data for characterization of antibodies used for NEUROD2 ChIP-Seq experiments.** In order to test for antibodies that work well in the immunoprecipitation (IP) technique, we over-expressed myc-tagged NEUROD2 in Neuro2A cell line, immunoprecipitated with one of three different NEUROD2 antibodies, and immunoblotted (IB) with a myc antibody. All three antibodies robustly immunoprecipitated overexpressed NEUROD2. s/n: supernatant. (PDF 128 kb) Additional file 2:
**In this file we provide the raw sequencing counts and number of peaks for each ChIP-Seq experiment with individual antibodies used in this study.** (XLSX 7 kb) Additional file 3:
**In this file we list the genome-wide** ***in vivo*** **NEUROD2 peak coordinates.** (XLSX 3195 kb) Additional file 4:
**In this file we list the genome-wide** ***in vivo*** **NEUROD2 target genes.** (XLSX 799 kb) Additional file 5:
**Confirmation of NEUROD2 binding to putative target genes identified by the ClosesetGene method.** NEUROD2 ChIP and GFP ChIP DNA were used as templates for qPCR analysis. Data is represented as the amount of enrichment in target genes in NEUROD2 ChIP DNA as compared to a negative control GFP ChIP DNA. NEUROD2 non-target genes did not display a significant level of NEUROD2 binding. (XLSX 9 kb) Additional file 6:
**In this file we provide the list of most significantly represented pathways among NEUROD2 target genes (http://david.abcc.ncifcrf.gov/home.jsp).** (XLSX 7 kb) Additional file 7:
**In this table we provide the NEUROD2 target genes that are differentially expressed in the SVZ-IZ region.** (XLSX 10 kb) Additional file 8:
**In this table we provide the NEUROD2 target genes that function in axon guidance pathways.** (XLSX 11 kb) Additional file 9:
**This table lists the sequences of all primers used in this study.** (XLSX 9 kb)
