# Supplementary material for: Genome-wide target analysis of NEUROD2 provides new insights into regulation of cortical projection neuron migration and differentiation
Source: BMC Genomics. 2015 Sep 5;16:681. doi: 10.1186/s12864-015-1882-9 (PMC4560887; doi:10.1186/s12864-015-1882-9)
Supplement: Additional file 1: — In this file we provide raw data for characterization of antibodies used for NEUROD2 ChIP-Seq experiments. In order to test for antibodies that work well in the immunoprecipitation (IP) technique, we over-expressed myc-tagged NEUROD2 in Neuro2A cell line, immunoprecipitated with one of three different NEUROD2 antibodies, and immunoblotted (IB) with a myc antibody. All three antibodies robustly immunoprecipitated overexpressed NEUROD2. s/n: supernatant. (PDF 128 kb) [file 12864_2015_1882_MOESM1_ESM.pdf]

Additional file 1. Immunoprecipitation with three NEUROD2 antibodies

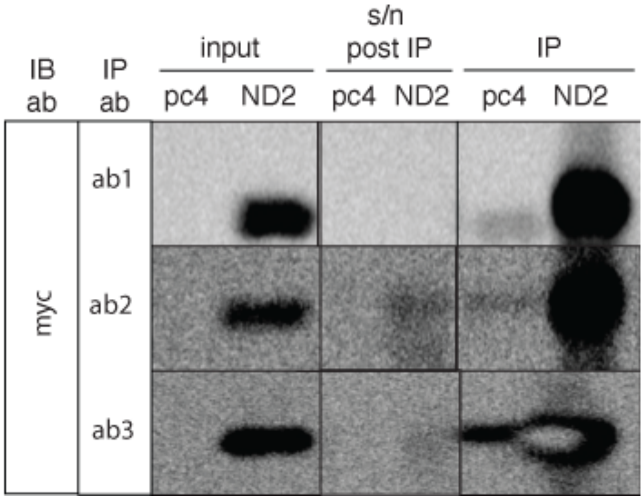

Neuro2A cells lines were transfected with Neurod2-myc or parent vector (pcDNA4). Immunoprecipitation was carried out as described in the *Methods* section. Western blotting was performed with a myc antibody. s/n: supernatent, IP: immunoprecipitation. All three antibodies successully immunoprecipitated NEUROD2-myc.

NEUROD2 antibodies	Specie	Brand	Catalog no.
ab1	mouse	Abcam	Ab168932
ab2	Rabbit	Abcam	Ab104430
ab3	Rabbit	Abcam	Ab109406
